# Hyaluronan Regulates Cell Behavior: A Potential Niche Matrix for Stem Cells

**DOI:** 10.1155/2012/346972

**Published:** 2012-02-12

**Authors:** Mairim Alexandra Solis, Ying-Hui Chen, Tzyy Yue Wong, Vanessa Zaiatz Bittencourt, Yen-Cheng Lin, Lynn L. H. Huang

**Affiliations:** ^1^Institute of Biotechnology, College of Bioscience and Biotechnology, National Cheng Kung University, Tainan, Taiwan; ^2^Research Center of Excellence in Regenerative Medicine, National Cheng Kung University, Tainan, Taiwan; ^3^Institute of Clinical Medicine, College of Medicine, National Cheng Kung University, Tainan, Taiwan; ^4^Advanced Optoelectronic Technology Center, National Cheng Kung University, Tainan, Taiwan

## Abstract

Hyaluronan is a linear glycosaminoglycan that has received special attention in the last few decades due to its extraordinary physiological functions. This highly viscous polysaccharide is not only a lubricator, but also a significant regulator of cellular behaviors during embryogenesis, morphogenesis, migration, proliferation, and drug resistance in many cell types, including stem cells. Most hyaluronan functions require binding to its cellular receptors CD44, LYVE-1, HARE, layilin, and RHAMM. After binding, proteins are recruited and messages are sent to alter cellular activities. When low concentrations of hyaluronan are applied to stem cells, the proliferative activity is enhanced. However, at high concentrations, stem cells acquire a dormant state and induce a multidrug resistance phenotype. Due to the influence of hyaluronan on cells and tissue morphogenesis, with regards to cardiogenesis, chondrogenesis, osteogenesis, and neurogenesis, it is now been utilized as a biomaterial for tissue regeneration. This paper summarizes the most important and recent findings regarding the regulation of hyaluronan in cells.

## 1. Hyaluronan Properties

Hyaluronan (also known as hyaluronate or hyaluronic acid, HA) is a nonsulfated linear polysaccharide present in the extracellular matrix of every vertebrate's tissue. It is a member of the glycosaminoglycan (GAG) family and is synthesized at the inner leaflet of the plasma membrane [1] as a large, unbranched polymer of repeating disaccharides of glucuronic acid and *N*-acetylglucosamine. It has a simple chemical structure but differs from other glycosaminoglycans in its high molecular weight, lack of sulfate groups, and absence of covalent attachment to core proteins [1]. It was assumed that its major functions were in joint lubrication, tissue homeostasis, and holding gel-like tissues together. However, the high molecular weight of hyaluronan enables it to acquire a viscous characteristic that goes beyond these functions. Indeed, hyaluronan coregulates numerous physiological processes including embryonic development, inflammation, tissue regeneration, cell migration, and proliferation. Some reports [2, 3] have also suggested that hyaluronan plays some role in cancer invasion and in the promotion of angiogenesis.

Many functions of hyaluronan depend upon its interaction with cell surface receptors, including cluster determinant 44 (CD44), receptor for hyaluronan-mediated motility (RHAMM), lymphatic vessel endothelial hyaluronan receptor (LYVE-1), hyaluronan receptor for endocytosis (HARE), liver endothelial cell clearance receptor (LEC receptor), and toll-like receptor 4 (TLR4) [1]. Although it is well established that hyaluronan-induced signaling occurs through receptor interactions, its signal transduction mechanism in cells has not been fully characterized.

Stem cells have the unique abilities of self-renewal and pluripotency. These characteristics therefore play essential roles in organogenesis during embryonic development and tissue regeneration. The potential utility of stem cells for tissue regeneration and treatment of many formerly incurable diseases has motivated scientists to discover the key influences of stem cell behavior. The identification of hyaluronan in many locations where stem cells are present raised the possibility that hyaluronan influences these potent cells. Indeed, emerging studies [4–6] have proven the veracity of this hypothesis, and hyaluronan is now being studied as a key component that may be manipulated to induce desired stem cell behaviors.

## 2. Hyaluronan Receptors and Signaling

Hyaluronan interacts with cell surfaces in at least two ways. It can bind cell surface receptors, hyaladherins, such as CD44, LYVE-1, HARE, layilin, TLR4, and RHAMM, to induce the transduction of a range of intracellular signals, either directly or by activating other proteins. Through signal-transducing receptors, hyaluronan influences cell proliferation, survival, motility, and differentiation and might have roles in cancer pathogenesis. The detail signaling mechanisms for each receptor is reviewed as follows.

### 2.1. HARE

HARE, also known as stabilin-2, is a transmembrane receptor protein that contains a C-type lectin-like HA-binding module, which enables binding and endocytosis of hyaluronan ligand. The function of this receptor has been regarded to be involved in the mediation of normal turnover process of hyaluronan and other GAGs, such as chondroitin sulfate, from the circulatory system. Hyaluronan is internalized via a clathrin-coated pit pathway, leading to hyaluronan degradation in the lysosome [7].

### 2.2. Layilin

Similar to HARE, layilin is a transmembrane protein homologous to C-type lectins, which is located at the membrane ruffle. It acts as a membrane docking site for talin and can also specifically bind hyaluronan [8, 9] through its lectin-like domain. Similar to CD44, layilin extracellularly binds HA and induces binding of cytoskeletal proteins such as talin and ERM complex through its cytoplasmic domain. After receiving the HA signal, layilin interacts with merlin, a protein of the ERM superfamily, at the amino terminus and modulates cell cytoskeletal structure [10].

### 2.3. RHAMM

RHAMM is a ubiquitous protein which is present in the nucleus, cytoplasm, and cell plasma membrane [11–13]. RHAMM can be alternatively spliced to produce molecules of different sizes. When the membrane-bound RHAMM interacts with hyaluronan, kinase activity of c-Src, FAK, or PKC is activated [13, 14]; the intracellular RHAMM domain is capable of binding kinase or cytoskeletal components [11], forming a cytoskeleton regulating complex and triggering cytoskeletal rearrangement. Disruption of hyaluronan-RHAMM interaction using either protein mutation or antisense mRNA treatment significantly reduces cell motility rate and formation of stable focal adhesions [14], supporting the model of RHAMM-mediated regulation of FAK activity. It has been reported that RHAMM binding of epiregulin, a novel ligand of EGFR, activates downstream tyrosine kinase-mediated autophosphorylation and regulates cell survival and proliferation of human cementifying fibroma [15] cells (HCF).

### 2.4. LYVE-1

LYVE-1 is a type I integral membrane protein containing a link domain, similar to the proteolytic hyaluronan binding domain of the Link protein superfamily [16]. LYVE-1 is identified as an endocytic receptor of hyaluronan, specifically expressed in the lymphatic endothelium. LYVE-1 has been widely used as a marker for investigation of lymph vessel structure in both benign and malignant prostate tissues and hence applicable in cancer-related studies. In HS-578T human breast cancer cells, adhesion to COS-7 cells was enhanced by overexpression of LYVE-1. This phenomenon was reduced by enzyme digestion treatment of HS-578T cell surface with bacterial *Streptomyces hyaluronidase* prior to adhesion experiment, suggesting that LYVE-1 enhances tumor cell adhesion through interaction with hyaluronan. A recent study pointed out that in human primary effusion lymphoma (PEL) tumor cells, when LYVE-1 and EMMPRIN/CD147 coexpressed with drug transporter protein, breast cancer resistance protein/ABCG2 (BCRP), EMMPRIN induced upregulation of BCRP, and LYVE-1 colocalized with BCRP on the cell surface, suggesting that LYVE-1 takes part in facilitating hyaluronan and EMMPRIN-mediated chemoresistance.

### 2.5. CD44

**Figure 1:**
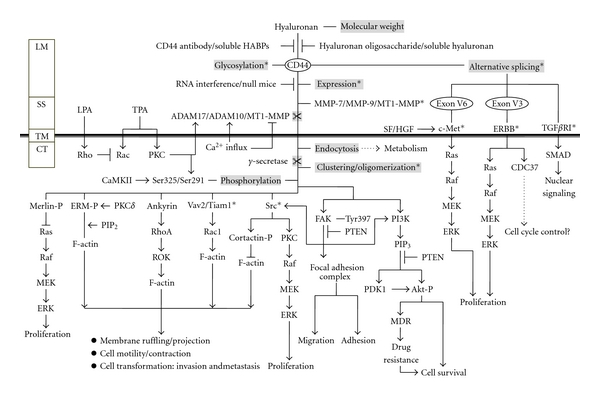
Signaling through CD44: hyaluronan binding to a receptor such as CD44 or RHAMM will cause a conformational change in the receptor. In the case of CD44, *γ*-secretase cleavage of the intracellular fragment can lead to phosphorylation of cellular components such as CaMK (calmodulin kinase). Clustering of the intracellular portion of CD44 with cellular proteins such as merlin, Src, and PKC leads to downstream activation of the Raf/MEK/ERK pathway, enhancing cell proliferation. Alternatively, activation with ankyrin, ERM, or Vav2/Tiam1 leads to F-actin activation and cytoskeletal rearrangement, membrane ruffling, and cell motility. Hyaluronan signaling also regulates cell migration and adhesion through interaction of PI3K and activation of the focal adhesion complex. PI3K activation by hyaluronan signaling, when incorporated with the Akt pathway, may also lead to MDR and cell survival.

CD44-mediated cell interaction with hyaluronan has been implicated in a variety of physiological events including cell-cell and cell-substrate adhesion, cell migration, cell proliferation, and hyaluronan uptake and degradation [17]. A proposed signaling pathway activated by the CD44 receptor is depicted in Figure 1. Hyaluronan is often bound to CD44 isoforms, which are ubiquitous, abundant, and functionally important cell surface receptors [18] in coordinating intracellular signaling pathways (e.g., Ca^2+^ mobilization [19], Rho signaling [20], PI_3_-kinase/AKT activation [21], NHE1-mediated cellular acidification [22], transcriptional upregulation, and cytoskeletal function [23]) and generating the concomitant onset of tumor cell activities (e.g., tumor cell adhesion, growth, survival, migration, and invasion) and tumor progression. CD44-hyaluronan interaction in MSC migration can be found in the rat MSC line Ap8c3 and mouse CD44^−/−^ or CD44^+/+^ bone marrow stromal cells. Adhesion and migration of MSC Ap8c3 cells [24] to hyaluronan was suppressed by anti-CD44 antibody and by CD44 small interfering RNA (siRNA). In some tumor studies, hyaluronan binding to tumor cells promoted Nanog protein in association with CD44 followed by Nanog activation and the expression of pluripotent stem cell regulators such as Rex1 and Sox2. Nanog also formed a complex with the signal transducer and activator of transcription protein 3 (Stat-3) in the nucleus leading to Stat-3-specific transcriptional activation and MDR1 (P-glycoprotein) multidrug transporter gene expression [25].

Scientists have long noticed superficial similarities between stem cells and cancer cells. We believe that CD-44 mediated cell interaction with hyaluronan will provide valuable new insights into previously understood aspects of solid tumor malignancy. In fact, human breast cancer cells strongly expressing CD44 along with low or no expression of CD24 effectively formed tumors. These cells possess the ability to differentiate, proliferate, and self-renew, comparable to normal stem/progenitor cells; hence, CD44^+^/CD24^low/-^ cells were considered to possess the stem/progenitor cell phenotype [26]. Later, this idea was confirmed when epithelial mesenchymal transition (EMT) traits were observed to correlate with the CD44^+^/CD24^low/−^ stem cell phenotype in human breast cancer [27]. Another marker, ALDH1, further divides the CD44^+^CD24^low/−^ cell population into 2 fractions, in which the ALDH1^+^CD44^+^CD24^low/−^ cells are highly tumorigenic [28]. Forced expression of CD24 in CD44^+^CD24^low/−^ cells resulted in decreased MEK/MAPK signaling, reduced cell proliferation, and enhanced DNA damage-induced apoptosis through attenuated NF*κβ* signaling [29]. In contrast, siRNA silencing of CD44-encoding genes in CD44^+^CD24^low/−^ cells enhanced sensitivity to doxorubicin, suggesting CD44 as a suitable target for treating cancer via gene therapy [30].

This new understanding of hyaluronan/CD44-mediated oncogenic signaling events may have important clinical utility and could establish CD44 and its associated signaling components (hyaluronan/CD44-mediated Nanog-Stat-3) as important tumor markers for early detection and evaluation of oncogenic potentials. This could also serve as groundwork for the future development of new drug targets that may inhibit hyaluronan/CD44-mediated tumor metastasis and cancer progression.

## 3. Hyaluronan Constitution in the Stem Cell Niche

After the term “niche” was first proposed in 1978 [31], important related studies in cell biology emerged, including a focus on the mysterious microenvironment that supports stem cells. The stem cell niche is not solely the location where these cells are present but involves the surrounding cellular components of the microenvironment and the signals emanating from the support cells [32]. This niche requires an environment that fosters a delicate balance between self-renewal and differentiation. Absence of this balance immediately triggers inappropriate differentiation. Indeed, a decrease in proliferation potential of bone-marrow-derived hematopoietic stem cells (HSC) has been observed in the absence of a “niche” environment [33]. Hematopoiesis, the process by which HSC differentiate into hematopoietic cells in order to generate different blood cell types, is greatly influenced by the surrounding microenvironment. The actual mechanism by which HSCs interact with the niche environment remains largely unknown; however, many studies have shown that the HSC niche is important for attracting and anchoring HSCs. Hyaluronan, being a critical component of the HSC microenvironment and widely distributed in mesenchymal tissue, is thought to take part in the postnatal hematopoietic niche since it is required for in vitro hematopoiesis [34]. Hyaluronan degradation leads to an arrest in HSC proliferation that is necessary for commitment to the maturation of hematopoietic cells [34].

In vertebrates, hyaluronan appears to regulate cell transformation and migration at several embryonic stages, as early as gastrulation. During embryogenesis, the accumulation and organization of hyaluronan plays a role in the epithelial-mesenchymal transition, a critical step in early embryogenesis for differentiation of pluripotent embryonic stem cells (ESC) to mesenchymal stem cells (MSC) for further formation of different tissues [35]. In general, endogenously produced hyaluronan contributes to differentiation of hESC to mesodermal lineage, but more specifically to hematopoietic cells [36]. The likely source of hyaluronan during early embryonic developmental stages is hyaluronan synthase (Has) 2 [37]. Many cardiac or skeletal development anomalies are due to inactivation or upregulation of hyaluronan synthetases. It seems that interaction of hyaluronan with ESC is fundamental to early embryonic development. Failure of this interaction results in abnormal tissue formation. Hence, hyaluronan has a central regulatory role during embryogenesis and morphogenesis [38, 39]. At later stages, Has1, Has2, and Has3 are expressed in both undifferentiated and differentiated hESC [36]. During embryonic development, hyaluronan mostly interacts with cells via the RHAMM and CD44 receptors [15, 34]. High-molecular-weight hyaluronan induces CD44 association with MEKK1 in epicardial cells to promote epithelial mesenchymal transition and differentiation [40]. However, more recent studies have observed that the HA-CD44 signaling pathway is largely associated with activation of ERK, by which the EGF receptor and downstream molecules such as Raf, MEK, and ERK1/2 are activated to promote cell proliferation [15]. ERK and its downstream molecules cyclin D1 and E2F also affect cell cycle progression [41].

In addition to collagen, alginate, and fibrin, hyaluronan is one of the extracellular matrix molecules being used to develop scaffolds for in vitro studies of stem cells, mainly due to its great abundance in the stem cell niche environment. Human ESCs may be maintained in an undifferentiated state by utilizing hyaluronan hydrogels [42], once more indicating the importance of the interactions provided by hyaluronan.

## 4. Influence of Hyaluronan on Stem Cell Behavior

MSCs are naturally sensitive to their environment, responding to chemical, physical, and mechanical features of their matrices or substrates, as well as the spatial/temporal presentation of biochemical cues [43]. Hyaluronan influence in behaviors such as adhesion, proliferation, differentiation, and migration occurs through several newly discovered genetic signaling mechanisms that involve binding to specific cellular receptors.

### 4.1. Effect of Hyaluronan on Mitochondrial Function

The effect of hyaluronan in cell behavior involves a direct influence in cell metabolic activity including bioenergetics. Few studies have underscored this phenomenon in stem cells; however, the first report suggesting an influence of hyaluronan on mitochondrial properties was obtained through studies of chondrocytes from osteoarthritis (OA) patients, who exhibit disrupted cellular behavior [44]. The fact that abundant levels of hyaluronan are present in normal and healthy joint areas and that these levels are dramatically diminished in OA patients [44] suggests that hyaluronan abundance may be a somewhat chondroprotective. Because oxidative stress, disrupted mitochondrial respiration, and mitochondrial damage promote aging, cell death, functional failure, and degeneration in a variety of tissues [45], including joint regions, it is possible that the putative chondroprotective role of hyaluronan may occur through the preservation of mitochondrial function. This was proven true when incubation of primary human chondrocytes from OA patients with hyaluronan significantly increased mtDNA integrity, improved ATP levels, and increased cell viability under normal conditions [44]. Moreover, hyaluronan ameliorated the negative effects of reactive oxygen and nitrogen species on mtDNA integrity, mtDNA repair, ATP production, and cell viability, all of which chondrocytes are exposed to during OA. Anti-CD44 antibody at saturating concentrations abolished the protective effects of hyaluronan, which suggests the mechanism is mediated by this receptor. CD44 promotes apoptotic resistance in colonic epithelium via a mitochondria-controlled pathway [46]. In addition, expression of CD44 in some cell types, such as stem cells, may provide the means to internalize hyaluronan by endocytosis, and one of the functions of internalized hyaluronan is to protect DNA from oxidative metabolism [47]. In this study, high-molecular-weight hyaluronan in culture medium prevented H_2_O_2_-induced H2AX phosphorylation in 2 cell types. In contrast, the effect of low-molecular-weight hyaluronan was somewhat less pronounced. Indeed, there is evidence that some glycosaminoglycans such as chondroitin-4-sulfate and hyaluronan inhibit lipid peroxidation caused by oxidative stress and thereby decrease inflammatory reactions mediated by oxidants [48]. These conditions mimic the situation in vivo when cells are growing within an intercellular matrix containing hyaluronan and are exposed to an exogenous oxidizing agent. This supports the proposition that one of the biological functions of hyaluronan is to provide protection against cellular damage caused by radicals produced by oxidation or ionizing radiation.

 Stem cells thus may have several diverse mechanisms to protect the integrity of their DNA, such as by maintaining low metabolic activity to ensure minimal oxidative damage, having a highly effective efflux pump that rapidly removes genotoxic agents from the cell [49], and possibly allowing internalization of hyaluronan, which protects DNA from oxidants. These mechanisms may be coordinated with each other and with cell cycle status. However, information is sparse concerning how exactly CD44 may affect mitochondrial function. Stem cell mitochondria have recently come under increased scrutiny because new information has revealed their role in numerous cellular processes, beyond ATP production and apoptosis regulation, suggesting mitochondria to serve as a cell fate or lineage determinant [50–52]. Thus, hyaluronan influence on mitochondrial properties may at least to some extent affect stem cell self-renewal and differentiation. Unfortunately, no ongoing studies have been published thus far.

### 4.2. Cell Migration

Observations made by a very successful research group in Boston in the beginning of the 1970s revealed that hyaluronan accumulation coincides with periods of cellular migration [53]. This event may occur through the physiochemical properties of hyaluronan or via direct interaction with cells. When hyaluronan is synthesized and released to the extracellular environment, its physiochemical characteristics of viscosity and elasticity contribute to local tissue hydration. This results in weakening of cell anchorage to the extracellular matrix, allowing temporal detachment to facilitate cell migration and division [54]. Chemically, this can be explained by the hyaluronan structure. In dilute solutions, where the domain that is occupied by each hyaluronan molecule expands because of mutual repulsion between the carboxyl groups inside its structure, a large volume will eventually be occupied with water trapped inside it. This property provides resilience and malleability to many tissues so that in hyaluronan-rich areas internal pressure may cause the separation of physical structures and create “highways” for cell migration; during fetal development, the migration path through which neural crest cells migrate is rich in hyaluronan [55]. Another example is the migration of mesenchymal cells into the cornea following increased hyaluronan deposition, hydration, and concomitant swelling of the migratory pathway [56]. Beyond the physiochemical interactions with hyaluronan, cells may be able to mediate, direct, and control their migration and locomotor mechanism through specific interactions via cell surface hyaluronan receptors. The principal cell surface receptors include CD44, RHAMM, and ICAM-1 [17]. RHAMM in particular forms links with several protein kinases associated with cell locomotion, for example, extracellular signal-regulated protein kinase (ERK), p125^fak^, and pp60^c-src^ [17]. Increased cell movement in response to hyaluronan can also be demonstrated experimentally in other cell types, and cell movement can be inhibited, at least partially, by degradation and/or blocking of hyaluronan receptor occupancy.

### 4.3. Cell Cycle and Proliferation

The formation and repair of mature hard tissue require cell proliferation. Hyaluronan levels have been shown to have a direct influence on this event. Cell proliferation is activated by hyaluronan, which increases volume and surface area for cell migration and cellular activities, and stimulates receptor-mediated events. Hyaluronan can form a pericellular coat, settle into a cell-poor space, and facilitate cell detachment from the matrix and mitosis in response to mitogenic stimulators such as preinflammatory mediators and growth factors [57]. Human fibroblasts synchronized with colchicine or cytochalasin showed increased hyaluronate synthesis at the time of cell rounding during mitosis but declined sharply as cells entered G_1_-phase and resumed the spread morphology [57]. It is reasonable to suggest that basal levels of hyaluronan synthesis during the G_1_, S, and G_2_ phases could be used for cell migration; during mitosis, synthase activity could be activated at all cellular contact areas to cause detachment and rounding. The high local concentration of hyaluronan causes release of endogenous growth factors and stimulates cell-cell interaction, resulting in faster cell proliferation during early stages of in vitro culture [58]. Although hyaluronan facilitates cell detachment, it has not been shown to possess direct mitogenic activity. However, by facilitating cell mitosis in response to mitogenic factors that are abundant during the early phases of tissue repair, hyaluronan may also have an important, although indirect role in cell proliferation [17]. 

Many reports have experimentally confirmed this idea, demonstrating the acceleration of stem cell proliferation by hyaluronan. In previous studies using mouse adipose-derived stem cells (mADSCs), we showed that supplementation of the culture medium with minute amounts of high-molecular-weight hyaluronan increases the growth rate of mADSCs at early passages, contributes to the extension of their lifespan with a marked reduction of cellular senescence during subcultivation, and prolongs their differentiation potential [4]. When mADSCs were cultured on a hyaluronan precoated surface, cell aggregates formed with a much more gradual growth profile. Hyaluronan-containing matrix seemed to be a poor attachment substratum for cell growth, as previously observed [59]. At later passages, aggregation limited propagation and contact inhibition, resulting in a slight increase of p16INK4a expression, consistent with previous reports [60]. Our study provided evidence that placenta-derived mesenchymal stem cells (PDMSCs) grown on a hyaluronan-coated surface are maintained in slow-cycling mode and that a prolonged G_1_ phase occurs through elevated levels of p27^kip^ and p130, which are responsible for suppressing cell entry into S-phase [6]. This in turn may be the main cause of reduced proliferation observed in stem cells cultured on hyaluronan-coated surfaces [4]. This line of evidence was confirmed by another research group who found that a high percentage of primary rat calvarial osteoblasts, in the presence of sulfated hyaluronan (HAS) derivatives, remained in G_1_ phase with a concomitant decrease in the number of cells in S and G_2_/M phase, eventually leading to a decrease in cell proliferation [61]. Recent studies have shown that prolonged G_1_ transit is associated with an increase in p27^Kip1^ [62]. Because p27 phosphorylation by cyclin E/Cdk2 is a prerequisite for its ubiquitination and degradation, it is interesting that in CD44-treated cells cyclin E/Cdk2 kinase activity is decreased, suggesting that CD44 might inhibit the ubiquitin-dependent proteolytic pathway of p27, leaving this molecule in an active form [62]. The specific signaling cascade that inhibits p27 degradation via CD44 remains to be characterized. However, it was speculated that blockage of the Ras pathway, which represents a principal force in driving the cell cycle [63], is involved in this process [64–66]. 

Hyaluronan thus appears to be effective in maintaining mADSCs in a proliferative state, delays senescence, and, more strikingly, directly influences cell proliferation. Different forms of hyaluronan supplementation may cause distinct proliferative behaviors in stem cells. Although the mechanism by which hyaluronan promotes or slows proliferation and preserves the differentiation potential of mADSCs merits further investigation, the addition of hyaluronan to a culture system may be a useful approach for expanding adult stem cells in vitro without losing their replicative and differentiation capabilities.

### 4.4. Cell Differentiation

In native tissue, MSCs reside in a defined microenvironment that regulates stem cell survival, self-renewal, and differentiation through growth factors, cell-cell contact, and cell-matrix adhesion. This occurs due to the direct influence of cell adhesion to their underlying biomaterials. Although soluble factors are potent regulators of stem cell differentiation, recent discoveries have underscored the importance of the physical and chemical characteristics of the matrices in determining stem cell fate [67]. Long-term culture of undifferentiated ESCs on a hyaluronan-coated surface instead of feeder layers yields pluripotency and differentiation characteristics similar to those of cells cultured on mouse embryonic fibroblasts (mEF) after 1 month of culture [68]. It seems that hyaluronan matrices act as a unique microenvironment for propagation of hESCs, likely due to the regulatory role of hyaluronan in the maintenance of hESCs in their undifferentiated state in vitro and in vivo. Indeed, in humans, the hyaluronan content is greatest in undifferentiated cells during early embryogenesis and decreases at the onset of differentiation [39]. It has been suggested that hESCs are able to take up and degrade hyaluronan through CD44 and thereby remodel hyaluronan matrices, a feature necessary for cell survival and migration [42]. After activation of several environmental cues that dictate the need for fully differentiated cells, hyaluronan supplementation may in turn induce faster cell attachment and enhance cell differentiation, possibly through improved cell-cell communication [33]. How hyaluronan may mediate this event is still unknown but has been confirmed by a new, engineered class of hyaluronan-based hydrogels that provide a natural extracellular matrix environment with a complex mechanical and biochemical interplay. The hydrogel induced osteoblast differentiation of MSCs without the use of osteogenic media. This most likely occurred through the enhancement of cell adhesion [33].

### 4.5. Multidrug Resistance

“Classic multidrug” resistance (MDR) is caused by increased drug export through ATP-dependent efflux pumps, such as multidrug-resistance proteins (MRPs), and other members of the ATP-binding cassette (ABC) transporter families [69]. The finding that hyaluronan stimulates cell survival signaling and that treatment of tumor cells with hyaluronidase increased the activities of various chemotherapeutic agents [70] led to the further investigation of the possible role of hyaluronan in drug resistance. Increased hyaluronan production stimulates drug resistance in drug-sensitive cancer cells, whereas disruption of endogenous hyaluronan-induced signaling suppressed resistance to doxorubicin, taxol, vincristine, and methotrexate [71]. Therefore, the effects of hyaluronan on cell-survival signaling might alter drug resistance. Although the antiapoptotic effect of hyaluronan probably contributes to these phenomena, it is also known that lipid products of PI_3_K, such as phosphatidylinositol-3,4-diphosphate, and phosphatidylinositol-3,4,5-triphosphate, directly mediate the function of ABC transporters that are involved in bile transport [72]. Because hyaluronan stimulates PI_3_K activity, it might also influence drug resistance by stimulating drug transport [2]. Thus, transmembrane pumps may induce MDR by decreasing the intracellular accumulation and retention of drugs. Of particular interest is a recent work [73] indicating that inhibitors of MDR block hyaluronan synthesis and secretion and that hyaluronan might be secreted through multidrug transporters. Another possible regulator of hyaluronan-mediated multidrug resistance is the extracellular-matrix metalloproteinase inducer (EMMPRIN). EMMPRIN also stimulates the production of hyaluronan in mammary carcinoma cells [74]. Consequently, EMMPRIN promotes cell-survival signaling and induces multidrug resistance in a hyaluronan-dependent manner. In previous studies, we provided evidence that PDMSCs grown on a concentrated hyaluronan-coated surface become doxorubicin-resistant and that the interaction between CD44 and hyaluronan is crucial for this drug resistance [5]. Hyaluronan-CD44 interactions upregulate expression of drug transporters, including MDR1 [75], MRP2 [75], and BCRP [76]. In addition, small fragments of hyaluronan may mediate multidrug resistance by binding to CD44 and promoting translocation of a specific transcriptional regulator (YB-1) of the multidrug resistance MDR1 gene. This event simultaneously induces P-glycoprotein, the product of the MDR1 gene, and a broad-spectrum multidrug efflux protein present in both cancerous and healthy cells. Previous studies have reported that MDR is an important regulator of stem cell commitment [77]. Thus, we suggest that P-glycoprotein induced by the hyaluronan-coated surface holds PDMSCs in a primitive state by enabling them to extrude molecules required for differentiation. To the best of our knowledge, no reports have been published on MDR acquisition in mesenchymal stem cells; therefore, our study was the first to show that a hyaluronan substratum induces PDMSCs to acquire MDR as a result of increased P-glycoprotein expression through CD44 signaling. These and previous observations [4, 6] lead us to suggest that hyaluronan may cause PDMSCs to become dormant, the natural state of stem cells, which is consistent with slow cycling and drug resistance [5].

## 5. Hyaluronan Regulates Tissue Morphogenesis

Hyaluronan plays an important role in many morphogenetic processes during vertebrate development. Interaction of hyaluronan with surrounding stem cells regulates cell differentiation or the fate of cells. After hyaluronan comes in contact with surrounding cells through receptors, hyaluronan degradation by proteolysis releases cell-associated factors which affect cell motility.

### 5.1. HA in Cardiogenesis

High-molecular-weight hyaluronan (HMW-HA) stimulated epicardial cells, during formation of coronary vasculature in embryonic development, promoted association of hyaluronan receptor CD44 with MEKK1 protein, induced MEKK1 phosphorylation, and activated the ERK-dependent and NF*κ*B-dependent pathways. Two methods of CD44 blockage decreased the HMW-HA-induced invasive response in epicardial cells [40]. Recent development of hyaluronan mixed esters of butyric and retinoic acids to improve the yield of cardiovascular stem cells revealed an enhancement in smad1, 3, and 4 gene expression and further upregulation of the cardiogenic gene Nkx-2.5 expression, which led to high cardiogenesis from stem cells [78].

### 5.2. HA in Osteogenesis and Chondrogenesis

The application of hyaluronan for bone regeneration is well known. In fact, the importance of hyaluronan in spine development was found in a hyaluronan synthase-2 (Has2) knockout mouse model [79]. Previous reports and our unpublished data have demonstrated the potential of hyaluronan in the enhanced chondrogenic and osteogenic differentiation potentials of mesenchymal stem cells via stimulated expression of specific target genes. These genes include chondrogenic markers such as sulfated glycosaminoglycans, SOX-9, aggrecan, and collagen type II; osteogenic markers alkaline phosphatase (AKP), osterix, runx2, and collagen type I [80–82].

### 5.3. HA in Neurogenesis

There is substantial evidence of hyaluronan participation in morphogenesis and neural cell differentiation in the central nervous system. Sulfated hyaluronan can increase proliferation of normal human astrocytes and differentiation through enhanced expression of connexin-26, -32, and -43 [83]. Hyaluronan-containing hydrogels provide a suitable environment for neural stem cell growth and differentiation [84, 85].

### 5.4. HA in Angiogenesis

Previous reports have shown that tumor growth and metastasis are dependent on neovascularization, and oligo-hyaluronan induces angiogenesis in vivo [3]. Oligo hyaluronan-induced proliferation of endothelial cells and tube formation during angiogenesis is mediated by CD44 signaling, followed by phosphorylation of Src, FAK, and ERK1/2 proteins, leading to upregulation of *c-jun* and *c-fos*. This phenomenon is reversed after silencing of CD44 with siRNA [86]. Hyaluronan has been tested for cell therapy in a limb ischemic mouse model and was shown to enhance angiogenesis and improve revascularization [55]. Our unpublished data demonstrated that in vitro culture of PDMSCs with high-molecular-weight hyaluronan slightly increased the expression of angiogenic markers, including KDR, CD31, and vWF.

## 6. Role of Hyaluronan and Stem Cells on Tissue Regeneration

Hyaluronan plays an important role in preservation of the normal extracellular matrix structure and induces neodermis at the wound bed [87]. It is extensively used as an important component of assorted categories of biomaterials. Hyaluronan, which supports stem cell interaction with extracellular matrix molecules [88], is capable of regulating the inflammatory chemokines, receptors, metalloproteinases, and tissue inhibitors that are necessary to form an efficient scaffold. Modulation of the expression of inflammatory factors (CXCL12, CXCL13, and CXCR5) in mesenchymal stem cells by hyaluronan contributes to regenerative processes [89]. In addition, fragmented hyaluronan stimulates inflammatory gene expression in various immune cells at injury sites via TLR4, TLR2, and CD44 [90]. This occurs through downregulation of the anti-inflammatory A2a receptor [91]. During leukocyte recruitment, hyaluronan interaction with CD44 activates various inflammatory cells, such as macrophages, through CD44-dependent signaling [92]. In contrast, low-molecular-weight hyaluronan induces dendritic cell maturation and promotes dendritic and endothelial cell release of proinflammatory cytokines such as TNF*α*, IL-1*β*, and IL-12 through TLR4 [93, 94]

The migratory, proliferative, and differentiation influences of hyaluronan in stem cells provide insights for the development of potent biomaterials for regeneration of full-thickness wounds (e.g., diabetic foot ulcer and burn wounds) through initial restoration of the dermal layer and a later application of differentiated local cells that may lead to faster and better regeneration [95]. For instance, regeneration of bone defects can be achieved by combining cells and osteogenic signals in a suitable scaffold. Combination of human bone-marrow-derived mesenchymal stromal cells and TGF*β* growth factor involved in chondrogenesis, on a commercially available hyaluronan biomaterial scaffold showed that cells were capable of proliferating and differentiating, forming a cartilage-like construct in vitro with increased expression of typical chondrogenic markers [88]. Combination of stem cells with bone morphogenetic protein-2 (BMP-2) in hyaluronan-based hydrogel has also been widely used for the same purpose [96]. 

Neural stem cell (NSC) therapy can be used for nerve and brain regeneration. Hyaluronan is the major extracellular matrix component of the adult central nervous system. Its presence alone is necessary for the differentiation of NSCs to neuronal cells in vitro [84]. Nevertheless, other reports have shown that using a 3D scaffold composed of HA, collagen, and the nerve growth factor neurotrophin-3 provided perfect nerve regeneration in vivo through differentiation of NSCs [97]. The efficacy of hyaluronan in several aspects of tissue regeneration is becoming better understood. This knowledge provides important insights into the therapeutic utility of stem cells in human disease.

## 7. Conclusion

The physiological role of hyaluronan, especially in regulation of cellular activities, may provide a potent therapeutic alternative with profound advantages. However, this hypothesis cannot be applied to clinical practice without a more complete biological understanding of its mechanisms. Different mechanisms of hyaluronan regulation in stem cells are now emerging but at a very slow pace. The possibility of maintaining stemness or even inducing differentiation by manipulation of hyaluronan concentration/molecular weight, or by targeting genes activated by hyaluronan, may be a start point in the race to find new therapies for fatal diseases. Still, many knowledge gaps need to be filled, opening a window for future research efforts.
